# Biomarkers in Systemic Lupus Erythematosus along with Metabolic Syndrome

**DOI:** 10.3390/jcm13071988

**Published:** 2024-03-29

**Authors:** Fernanda Isadora Corona-Meraz, Mónica Vázquez-Del Mercado, Flavio Sandoval-García, Jesus-Aureliano Robles-De Anda, Alvaro-Jovanny Tovar-Cuevas, Roberto-Carlos Rosales-Gómez, Milton-Omar Guzmán-Ornelas, Daniel González-Inostroz, Miguel Peña-Nava, Beatriz-Teresita Martín-Márquez

**Affiliations:** 1 Multidisciplinary Health Research Center, Department of Biomedical Sciences, University Center of Tonala, University of Guadalajara, Guadalajara 45425, Jalisco, Mexico; alvaro.tovar@academicos.udg.mx (A.-J.T.-C.); roberto.rosales@academicos.udg.mx (R.-C.R.-G.); milton.guzman@academicos.udg.mx (M.-O.G.-O.); 2 Department of Molecular Biology and Genomics, Institute of Rheumatology and Musculoskeletal System Research, University Center of Health Sciences, University of Guadalajara, Guadalajara 44340, Jalisco, Mexico; dravme@hotmail.com (M.V.-D.M.); flavio.sandoval@academicos.udg.mx (F.S.-G.); jesus.robles@academicos.udg.mx (J.-A.R.-D.A.); dgi-17@hotmail.com (D.G.-I.); miguel.pena4206@alumnos.udg.mx (M.P.-N.); 3 Rheumatology Service, Internal Medicine Division, Civil Hospital of Guadalajara “Dr. Juan I. Menchaca”, Guadalajara 44340, Jalisco, Mexico; 4 Academic Group UDG-CA-703, “Immunology and Rheumatology”, University Center of Health Sciences, University of Guadalajara, Guadalajara 44340, Jalisco, Mexico

**Keywords:** MetS, SLE, adipokines, CVD, microRNA

## Abstract

Metabolic syndrome (MetS) is a group of physiological abnormalities characterized by obesity, insulin resistance (IR), and hypertriglyceridemia, which carry the risk of developing cardiovascular disease (CVD) and type 2 diabetes (T2D). Immune and metabolic alterations have been observed in MetS and are associated with autoimmune development. Systemic lupus erythematosus (SLE) is an autoimmune disease caused by a complex interaction of environmental, hormonal, and genetic factors and hyperactivation of immune cells. Patients with SLE have a high prevalence of MetS, in which elevated CVD is observed. Among the efforts of multidisciplinary healthcare teams to make an early diagnosis, a wide variety of factors have been considered and associated with the generation of biomarkers. This review aimed to elucidate some primary biomarkers and propose a set of assessments to improve the projection of the diagnosis and evolution of patients. These biomarkers include metabolic profiles, cytokines, cardiovascular tests, and microRNAs (miRs), which have been observed to be dysregulated in these patients and associated with outcomes.

## 1. Introduction

Metabolic syndrome (MetS) is considered a noncommunicable disease in which the individual presents with three or more risk factors, including elevated blood pressure, large waist circumference, hyperglycemia, hypertriglyceridemia, low levels of high-density cholesterol (HDL-c), insulin resistance (IR), and increased blood pressure [1]. MetS is strongly associated with obesity and adiposity, determinants that resemble the phenotype of obesity-related IR, vascular stiffness, and endothelial dysfunction [2]. MetS is associated with cardiovascular disease (CVD), and one of the most critical etiological backgrounds of this pathology lies in the persistent metabolic alterations produced by adipose tissue dysfunction, which releases inflammatory cytokines, bioactive products such as adipokines, gaseous messengers, and microvesicles that alter the attached tissue phenotype and create reactions and molecules that are detectable at the systemic level [3,4].

The worldwide prevalence of MetS differs between populations due to different lifestyles. One of the most important modifiable factors is consuming a Western-pattern diet, characterized by processed and refined foods, red and processed meats, foods with added sugar and saturated and trans fats, and a low consumption of vegetables, fruits, nuts, and whole grains [5,6]. The prevalence of MetS differs between sexes, with females being the most affected [2,7,8,9].

On the other hand, immune–metabolic interactions observed in MetS have been associated with the development of autoimmune diseases, such as rheumatoid arthritis, ankylosing spondylitis, and systemic lupus erythematosus (SLE), among others [10].

SLE is the archetypical pathology of autoimmune diseases characterized by a wide range of clinical manifestations caused mainly by a complex interplay of environmental, hormonal, and genetic factors and the hyperactivation of immune cells [11].

In SLE, the breakdown of immune tolerance is essential for activating autoreactive B cells, consequently producing self-reactive antibodies. Autoantibodies such as anti-Smith (anti-Sm), anti-double-stranded DNA (anti-dsDNA), and anti-ribonucleoprotein (anti-RNP) are considered as hallmarks in SLE; nevertheless, these autoimmune diseases show a broad spectrum of more than 200 autoantibodies [12,13].

The mechanisms by which the autoantibodies cause tissue damage are mainly related to the accumulation of immune complexes, cytotoxicity, reactivity with autoantigens on the apoptotic cell surface, cell surface binding, and penetration into living cells [14].

During the clinical course of the disease, a wide range of organs may be affected, including skin, joints, lungs, heart, kidneys, hematological cells, and vascular and brain systems [12].

Regarding the vascular system, it has been documented that approximately 50% of SLE patients present with lupus vasculitis (LV), which mainly involves small- and medium-sized vessels. LV has many clinical forms that depend on the affected vessels and the site involved. The proposed pathogenic mechanism of LV is related to a complex interaction among vascular endothelium, immune cells, and their products, such as cytokines and autoantibodies, among which are antiphospholipid antibody (aPL) positivity, anti-endothelial cell antibodies, antineutrophil cytoplasmic antibodies (ANCA), and anti-dsDNA [15].

SLE patients are prone to suffer from cardiovascular diseases (CVDs) due to significant metabolic alterations in several pathways (i.e., lipoprotein metabolism) that might contribute to CVD pathology [16]. Indeed, a high prevalence of MetS has been reported in cohorts of SLE patients and is associated with increased disease activity, inflammation, and organ damage. MetS is recognized as a common comorbidity component of autoimmune diseases, where obesity has been associated with a low health-related quality of life in SLE patients [17].

Dietary intake and systemic metabolism are associated with the differentiation of the immune system. In this sense, it has been observed that the rates of autoimmunity are increasing in parallel with MetS, supporting the hypothesis that diet-induced obesity exacerbates autoimmune manifestations [18]. The activation of the immune system is necessary to initiate the production of interferon type I (IFN-I) and self-antigen-specific autoantibodies [19].

In experimental studies, obesity is related to the production of IFN-I by expanding plasmacytoid dendritic cells (pDCs) in visceral adipose tissue (VAT). Consequently, IFN-I impaired the number and function of T regulatory (Treg) cells, which could promote an autoimmune inflammatory microenvironment [17].

Since proinflammatory cytokines are a common underlying mechanism involving SLE and obesity, MetS probably triggers and contributes to overall inflammation, oxidative stress, and endothelial dysfunction [20].

Due to similarities in specific molecules’ expression in SLE and MetS, it is relevant to identify predictive biomarkers that contribute to an early and timely diagnosis in conjunction with the clinical assessment.

## 2. Metabolic Dysregulation in SLE and Its Biomarkers

Reports have revealed that the prevalence of MetS in SLE patients in individual studies ranges between 3.3 and 45.2% [20]. In this regard, it has been reported that SLE patients exhibit a profound alteration in lipoprotein metabolism pathways characterized by an increase in serum levels of total cholesterol, triglycerides (TG), apolipoprotein B (ApoB), and LDL-c and reduced HDL-c and apolipoprotein A1 (ApoA1) [16,21].

Moreover, it has been shown that, in SLE, the values of oxidized and dysfunctional HDL-c increase, contributing to the development of atherosclerosis and promoting an inflammatory response of macrophages through the activation of nuclear factor kappa-light-chain-enhancer of activated B cells (NF-ĸB) and the formation of neutrophil extracellular traps (NETs) and lipoprotein oxidation [22].

Although lipid profile is a crucial standard for assessing CVD risk in SLE patients, glucose and insulin levels are essential for assessing IR, a clinical state characterized by reduced insulin efficacy associated with increased insulin release and elevated blood concentration in an attempt to obtain an effective response to circulating glucose levels [23].

MetS patients are prone to develop IR, and the increment of fat mass could result in adipose tissue inflammation, a source of proinflammatory cytokines that can potentiate systemic inflammation [24]. For instance, tumor necrosis factor-alpha (TNF-α) is considered a central proinflammatory cytokine studied in IR that modulates the activity and expression of enzymes blocking insulin function [25]. High levels of TNF-α have been found in SLE patients with active lupus, demonstrating the enhanced risk of IR in these patients [26].

On the other hand, insulin levels and the homeostasis model assessment-estimated insulin resistance (HOMA-IR) index are increased in patients with SLE and SLE with MetS compared to healthy controls [27]. Studies in the literature have shown that SLE patients manifest deficient insulin secretion and a greater risk of IR. For instance, Petri et al. found that 7% of SLE patients present with type 2 diabetes mellitus (T2D), and 10% had glucose intolerance, showing that T2D may be more frequent in these patients than is estimated [28]. El Magadmi and colleagues found hyperinsulinemia and reduced insulin sensitivity in a cohort of SLE patients, where 18% had MetS [29]. For their part, Contreras et al. noticed that the increased waist circumference, higher acid uric levels, and longer duration of hypertension are factors associated with IR in SLE patients [30].

Regarding the metabolic disturbance that can be detected in SLE, Tang et al. recently found that, by analyzing metabolite profiles using mass spectrometry techniques with multiple reaction monitoring, SLE patients show alterations in fatty acid and phospholipid catabolism and elevated levels of pyroglutamic acid and L-phenylalanine [31].

Considering that, in SLE, the production of autoantibodies is a hallmark of this autoimmune disease, it has been noticed that specific autoantibodies contribute to atherosclerosis in pathology, such as anti-HDL-IgG related to induce LDL to enter the endothelial cells (EC) and anti-apolipoprotein A1 (ApoA1)-IgG, which induces the activation of NF-kB and favors the expression of inflammatory factors, among which are TNF-α, interleukin-6 (IL-6), interleukin-8 (IL-8), C-C motif chemokine ligand 2 (CCL2), and metalloproteinase 9 [32].

Furthermore, it has been observed that antibodies against lipoprotein lipase (anti-LPL) have been associated with increased levels of TG, Apo-E, and Apo-B in patients with SLE [33]. In addition, the production of autoantibodies such as aPL that are anticardiolipin antibodies (aCL) and anti-β2-glycoprotein I antibodies (anti-β2GPI) could increase endothelial injury and promote the induction of the proinflammatory endothelial phenotype interacting with endothelial cells (EC) [32].

At this point, we consider it essential to illustrate that the differences in affinity of antibody and antigen interactions are discriminated by the fragment crystallizable region receptor (FcR) that induces different signals at the molecule level, resulting in various immunological reactions [14]. For instance, IgG isotype antiphospholipid antibodies display a more predictive value of the vascular manifestation than IgM, while IgA aPL can be more predictive for vascular events than IgM [34].

## 3. Cytokines and Adipokines in SLE along with MetS

Cytokine production is closely related to the immune system response. Elevated levels of proinflammatory cytokines have been observed in SLE and MetS. Whereas one principal goal in SLE cytokine profile analysis is to identify cytokines as biomarkers that can be used to identify disease status and predict flares, in MetS, the analysis of molecules related to metabolism regulation, such as adipokines, is essential.

In obesity, the adipose tissue expansion is a primary source of proinflammatory cytokines. During homeostatic conditions, the adipose tissue-resident immune cells produce anti-inflammatory cytokines such as interleukin 4 (IL-4), IL-5, IL-10, IL-13, and transforming growth factor beta (TGF-β). The adipose tissue macrophages (ATM) are polarized to an M2 phenotype and enrich the adipose immune system together with other immune cells such as regulatory invariant natural killer T cells (iNKT), type 2 innate lymphoid cells (ILC2), regulatory T cells (Treg), and eosinophils, among others [35]. Meanwhile, in obesity, saturated fatty acids induce the ATM to polarize to an M1 phenotype, producing IL-6, TNF-α, IL-12, and IL-1β, among other inflammatory factors contributing to the impairment of insulin signaling and promoting IR [36].

Since obesity and overweight are identified risk factors for the development of metabolic disturbances in SLE, quantifying proinflammatory cytokines results in utility for its diagnosis and management. Different studies on SLE have determined increased IL-6 levels and their association with disease activity. Ding et al. performed a meta-analysis of 24 studies that showed serum IL-6 levels in SLE patients are higher than those in healthy controls and correlate with SLE activity [37]. Furthermore, IL-6 has also been associated with metabolic disorders such as MetS and T2D, in which elevated levels of IL-6 have been observed in adipose tissue [38].

On the other hand, TNF-α is a proinflammatory cytokine detected in high concentrations in SLE and correlates with disease activity and lupus nephritis. However, TNF-α has been found as a mediator of inflammation and regulator of autoimmunity, exerting in SLE a dual role [39]. Regarding the role of TNF-α in MetS, some reports indicate that it acts as an essential inducer of atherosclerotic plaques by driving the expression of adhesion molecules and favoring the activation of immune cells within the arterial wall [40]. Moreover, it is well documented that TNF-α promotes lipolysis, increases free fatty acid (FFA), and influences metabolic dysregulations such as IR. Furthermore, TNF-α can inhibit insulin-stimulated tyrosine kinase activity of the insulin receptor and substrate by inducing serine phosphorylation and producing IR [41].

On the other hand, it is known that adipocytes are not considered energy storage cells only; they are active producers of a specific type of cytokines called adipokines, which exert endocrine, paracrine, and autocrine effects [19]. Adipocytes play a crucial role in energy homeostasis regulation. The mechanism by which they accomplish this is through the secretion of adiponectin, leptin, resistin, IFN-I, TNF-α, interleukin 1 (IL-1), IL-6, and plasmin activator inhibitor type 1 (PAI-1), which are cytokines that take part in cellular intercommunication and act as master regulators of inflammation and metabolism that could influence the metabolic dysregulation in SLE and MetS [5,42].

In an obese state, an imbalance of adipokines promotes a low-grade proinflammatory state, resulting in IR and vascular dysregulation [43]. In addition to the above, it is known that in an autoimmune context, adipokines are involved in inflammatory pathways that affect a wide range of cell types, influencing systemic inflammation, chronically damaging tissues and organs, and impacting quality of life. In order to consider adipokines as biomarker candidates for SLE and MetS, we will describe those mainly associated with both pathologies.

Leptin is the main adipokine produced by adipocytes, whose concentrations positively correlate with adipose tissue mass. Leptin is involved in low-grade inflammation due to overweight and obesity and is considered a proinflammatory adipokine [44]. In SLE patients, leptin is highly produced; however, it does not correlate with disease activity [45].

Hyperleptinemia and leptin resistance are closely associated with obesity and T2D, and lower leptin concentrations in circulation are correlated positively with improved insulin sensitivity, lipid metabolism, lower adiposity, and inflammation [23].

Adiponectin is an adipokine that exerts anti-inflammatory effects. It is a complex molecule produced by adipocytes of the white adipose tissue (WAT), and its concentrations are inversely correlated with body mass index (BMI). Some of adiponectin’s functions are fatty acid biosynthesis and the inhibition of gluconeogenesis in the liver. It has been shown that, under systemic inflammation, adiponectin levels are modified, so an increase or decrease in its concentrations may be associated with pathophysiological conditions [44].

In SLE, higher adiponectin levels correlate positively with SLE severity and negatively with IR; however, when studies evaluate causal effects between circulating adiponectin levels and SLE, there are no relationship between circulating adiponectin levels and SLE risk [46].

Resistin is a cysteine-rich peptide hormone discovered in adipose tissue of rodents and detected in human peripheral blood mononuclear cells. Its functions are related to promoting immune response to inflammatory processes and enhancing heart, liver, and kidney diseases. In rodents, it has been found that WAT resistin secretion is associated with BMI and IR; in humans, the role of resistin in IR and T2D is controversial [47].

Adipsin is an adipokine mainly synthesized and secreted from WAT and exerts its functions by modulating glucose and lipid metabolism. Clinical data associate adipsin levels with BMI and visceral adipose tissue, whereas molecular assays reported that adipsin increases lipid accumulation and adipocyte differentiation through peroxisome proliferator-activated receptor (PPAR-γ) induction and complement component 3a (C3a) release [48]. Adipsin levels were found in higher concentrations in autoimmune diseases. However, further studies are needed to assess the association of adipsin with SLE [18].

Chemerin is an adipokine and chemoattractive protein that promotes inflammation, contributes to adipogenesis and glucose metabolism, and correlates with MetS [49]. In SLE, chemerin may serve as a marker of lupus nephritis (LN), where its circulating levels correlate with renal function [50].

Visfatin, also known as nicotinamide phosphoribosyl transferase (Nampt) was discovered as an insulin-mimetic adipokine produced mainly by visceral and subcutaneous adipose tissue, liver, immune cells, skeletal muscle, brain cells, cardiomyocytes, and renal glomeruli, among others. Its main functions are increasing glucose uptake by peripheral tissues, decreasing gluconeogenesis and glucose release, and stimulating the insulin cascade. In obesity, increased visfatin levels are related to a regulatory response to maintain glucose in stable levels; however, if the threshold is exceeded, visfatin is associated with inflammation and T2D, IR, CVD, and renal damage [51,52].

The role of visfatin in SLE is strongly associated with LN in humans and with pulmonary vasculitis and alveolar hemorrhage in experimental lupus models [53,54].

Omentin-1 is an adipokine expressed in higher levels in visceral adipose tissue; its concentrations decrease in overweight and obesity and increase after weight loss. Its biological activity is related to the enhancement of insulin-stimulated glucose uptake in human adipocytes [55]. Plasmatic omentin-1 levels were detected in differential expression between SLE patients with and without LN, suggesting that this adipokine could be employed as an auxiliary index [56].

Table 1 summarizes the results of different analyses in which adipokine serum or plasma levels were quantified in SLE and MetS studies.

## 4. Cardiovascular Clinical Assessments in SLE

Systemic inflammation is associated with the disease progression, and metabolic dysregulation in SLE can accelerate cardiovascular complications. The proinflammatory response is caused not only by the autoimmune underlying condition but also by the adipose tissue, which, if sustained, will magnify the risk of developing other metabolic and cardiovascular diseases.

Metabolic dysregulation is part of the deteriorating clinical status of a patient with SLE due to the increased risk of CVD, which, according to several studies, is higher than in the general population [10]. Esdaile and colleagues noticed that SLE patients present with a >7-fold higher risk of coronary heart disease (CHD) or stroke and 17-fold risk of having severe CHD death. Nevertheless, traditional Framingham risk factors cannot fully explain the significant CHD risk increase and, therefore, could be multifactorial due to a proatherogenic lipid profile, immune dysregulation and inflammation, side effects of treatment, and microvascular dysfunction [63,64]. Subsequently, in the Hopkins Lupus cohort, Magder and Petri et al. described that patients with SLE have a risk of cardiovascular events 2.66 times higher than expected in the general population with similar levels of traditional risk factors [65].

Oliviera et al. suggested that SLE patients should be assessed with classical CVD-related risks and disease activity parameters, such as aberrant adaptive immune response, proinflammatory cytokine signaling, elevated IFN-I, dysregulated NET formation, dysfunctional HDL-c, and oxidative stress [66]. Likewise, to observe any increased CVD risk, the European Alliance of Associations for Rheumatology (EULAR) recommendations state the measurement of traditional and disease-associated cardiovascular risk factors as necessary. For example, blood pressure (<130/80 mm/Hg), urine protein/creatinine ratio (>500 mg/g), QRISK3 assessment, and the American College of Cardiology/American Heart Association risk equation, although it is recognized that further studies are needed to validate cut-off points in this population. In addition, risk scores for the systemic lupus erythematosus disease activity index (SLEDAI), lupus anticoagulant, and C3a measurements are recommended [66].

Regarding the evaluation of atheroma plaque formation and atherosclerosis, it is considered essential to measure coronary artery calcifications and carotid intima-media thickness by techniques such as pulse wave velocity (PWV) or endothelium-dependent flow-mediated vasodilatation (ED-FMD), which are considered biophysical markers of endothelial dysfunction [67].

In a meta-analysis performed for SLE, the patients presented significantly higher PWV than controls; these results were associated with BMI and disease duration [68]. On the other hand, in studies performed in brachial artery ED-FMD (baED-FMD), SLE patients had lower baED-FMD than controls, reflecting impaired endothelial function [67].

Analyses carried out in this regard performed on SLE patients showed that those patients formed 2.0 times more atherosclerotic plaques in the carotid and femoral arteries than patients with rheumatoid arthritis and T2D and presented with 32% more atherosclerosis in the carotid compared to healthy controls [69]. In relation to the data previously shown, it has been determined that the risk of myocardial infarction in SLE is 2 to 3 times higher than in the general population [70].

To obtain an accurate diagnosis, other diagnostic tools, such as magnetic resonance imaging (MRI), must be considered. For instance, Mavrogeni et al. detected by MRI 27.5% silent cardiac abnormalities/past abnormalities such as myocarditis, myocardial infarctions, or vasculitis [71]. It is necessary to consider that antiphospholipid syndrome (APS) adds significant inflammatory thrombotic and atherosclerotic risks since there is an increased endothelial risk due to decreased endothelial nitric oxide synthase. As a result of APS, the increased production of nitric oxide, proinflammatory cytokines, adhesion molecules, and reactive oxygen species (ROS) and the activation of monocytes and the classical complement pathway are generated, further contributing to vascular inflammation [72].

Likewise, the use of glucocorticoids has been associated with atherosclerosis, hypertension, T2D, hypercholesterolemia, and CVD, even with the use of doses lower than those recommended. Although their use produces potent anti-inflammatory effects by reducing B and T lymphocyte activity, prostaglandin synthesis, and cyclooxygenases, it was observed that one-third of SLE patients are resistant to this treatment, probably due to modifications in the glucocorticoid receptor [65,73].

On the other hand, hydroxychloroquine (HQC) has been used to treat inflammatory diseases such as SLE, other autoimmune and neoplastic disorders, and metabolic and infectious diseases. When HQC is used in patients with SLE, some signs and symptoms, such as fatigue, skin rashes, joint pain, mouth ulcers, and arthralgia, improve and inhibit the production of inflammatory cytokines [74,75]. In addition, a decrease in aortic stiffness, systemic vascular resistance, and increased elasticity of the large arteries has been noticed; therefore, HQC therapy in SLE exerts a protective effect on coronary artery disease [76,77].

One of the proposed HQC action mechanisms is the reduction in ROS production, which decreases oxidative stress and improves the endothelial function. On the other hand, it has been observed that HQC increases LDL-c receptor expression and decreases serum cholesterol and TG concentration. At the same time, HQC inhibits cytokine production, apoptosis, and autophagy. According to the EULAR recommendations, HQC is classified as one of the disease-modifying antirheumatic drugs (DMARDs), and its use is widely recommended [74,76].

## 5. MicroRNAs in SLE with MetS Background

About the etiopathogenesis of SLE, it is currently known that both genetic and epigenetic factors may contribute to the development of autoimmunity. Nevertheless, it is also accepted that microRNAs (miRs) could be considered valuable biomarkers in diagnosis, treatment response, and general disease monitoring. The miRs have been evaluated from different perspectives, and, at a certain point, the large number of miRs that have been reported becomes overwhelming; however, between SLE and MetS, the associations made punctually between both pathologies are rare.

The miRs, typically known as non-coding regulatory RNA, are a class of small RNAs (about 22–25 nucleotides) that exert a crucial role in regulating the expression of its target genes at the post-transcriptional level [61]. It has been observed that miRs control the immune system as epigenetic regulatory elements regulating cellular development and differentiation [78]. As for miR biogenesis, the process is known to be as follows: miR genes are transcribed to generate an extended primary transcript (pri-miRNA) and processed to generate pre-miRNA in the nuclei. After being exported to the cytoplasm, the pre-miRNAs are processed to mature miRs by RNase III Dicer and then loaded into Argonaute (Ago) to form the RNA-induced silencing complex (RISC) effector, which represses translation [79]. Thus, miRs are considered an essential contributor to regulating the genes involved in the immune response because the RISC can silence messenger RNA at the pre-translational, co-translational, and post-translational levels, regulating 40% of the genes that control the differentiation of immune cells, their functions, and autoantibodies’ production [80].

The miR action is produced not only in the cells where they are synthesized but also can be transported to other target cells and found in the peripheral circulation. It also acts as a soluble marker sensitive to general health conditions. Furthermore, the most studied miRs in SLE are regulated by the expression of immune stimuli, like the presence of antigens, toll-like receptor (TLR) ligands, and inflammatory cytokines [81].

In SLE, NF-kB and IFN-I are deregulated pathways that interfere with inflammation. This pathway could be regulated by miR-146a and miR-155, which show potential for inhibition in the translation of IFN pathway signaling proteins such as TNF receptor-associated factor 6 (TRAF6), IL-1 receptor-associated kinase 1 (IRAK1), IFN regulatory factor 5 (IFN-5), and signal transducer and activator of transcription (STAT1) [82]. In a study by El-Akhras and colleagues in patients with SLE, they found that miR-146a was significantly increased in peripheral blood mononuclear cells (PBMC) and correlated positively with IL-6; based on these findings, they considered it a marketable marker for this autoimmune disease [83]. Additionally, Li et al. found in SLE patients that exosomal miR-155 is upregulated, whereas exosomal miR-146a was downregulated in SLE patients [84].

Regarding metabolic disturbances, it was observed that miR-146a is downregulated in obesity and correlates negatively with IL-6, TNF-α, and CD36 [85].

Another miR implicated in SLE pathology is miR-124, which modulates TLR-4-induced cytokine production by targeting signal transducers and STAT3 to decrease IL-6 production and TNF-α release. Zhang and colleagues found that miR-124 is downregulated in LN and could be considered as a significant diagnostic marker [86]. In addition, Yan et al. noticed that circulating miR-124-3p and miR-377-3p were significantly upregulated in SLE, where miR-124-3p is associated with antiC1q and C3, and miR-377-3p was determined as an independent predictor [87]. Concerning the influence of miR-124 in metabolic disorders, studies have revealed that miR-124 was highly expressed in T2D patients and related to lipid and glucose metabolism [88].

MiR-143/145 are intronic miRs expressed in endothelial cells that act in the differentiation and proliferation of vascular smooth muscle cells (VSMC) in the kidney; miR-145 is expressed in epithelial cells of proximal convoluted tubules and VSMC of renal vessels of children with LN, where its lower expression levels are related with the increase in vascular damage [81]. Moreover, increased levels of miR-143/145 in serum have been proposed as a potential diagnostic marker of SLE [89]. In T2D patients, miR-143 was detected in higher expression and associated with hypertension, increased body weight, glucose homeostasis, and impaired insulin activation [90].

Studies have observed that the miR-145 previously described acts as a positive regulator of the adipogenesis process and is related to the increase in adipose tissue/adipocytes in humans and rodents. Moreover, miR-145 promotes TNF-α secretion and lipolysis via NF-kB in vitro, showing their role in inflammation and cellular differentiation [91].

On the other hand, miR-125a negatively regulates the CCL5 upon activation of normal T cell expressed and secreted (RANTES); this proinflammatory cytokine is elevated in SLE and MetS [80].

Notably, the various treatment regimens for SLE can be considered a factor that may influence the expression of miRs and may be considered a biomarker of monitoring treatment response. For instance, Cecchi and colleagues observed that in SLE patients, rituximab affects the expression of circulating miRs, identifying a panel of five miRs, including miR-149-3p, miR-125b-5p, miR-199a-5p, miR-106b-3p, and miR-124-3p, as a potential target associated with proinflammatory cytokines and immune receptors [92]. These remarkable findings in the expression changes in miRs before and after treatment provide valuable information for future immunomodulation and diagnostic therapies of SLE.

## 6. MetS and SLE: A Harmful Relationship

**Figure 1:**
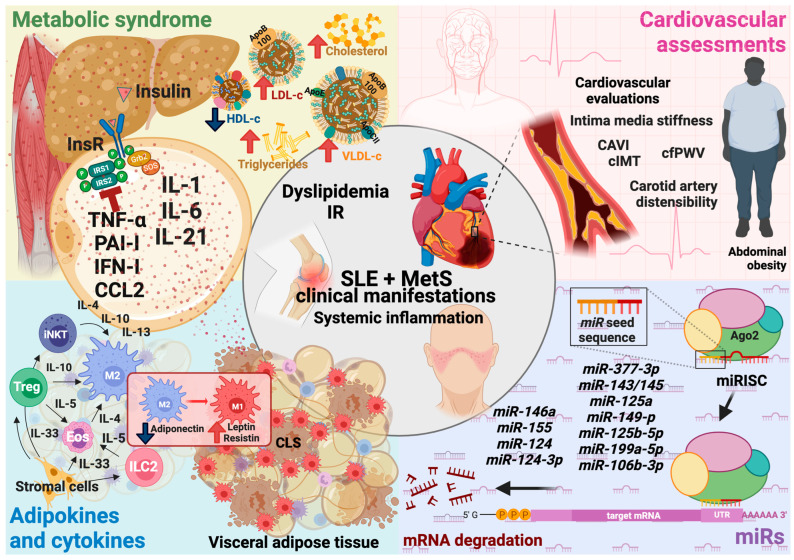
Systemic inflammation is the cornerstone where both pathologies converge and participate in the origin of clinical manifestations, development, and deterioration. Adipose tissue homeostasis becomes dysregulated as abnormal accumulation, and redistribution of body fat occurs during overweight and obesity due to chronic positive energy balance. This process modifies the immune cell population along with the T_H_2 cytokine microenvironment. It overcomes angiogenic and tissue remodeling capabilities to overcome adipocyte hypoxia and oxidative and concomitant endoplasmic reticulum stress, leading to proinflammatory adipokine and cytokine turnover. The resulting decrease in adiponectin and increase in proinflammatory adipocytokines and immune cells locally interfere with insulin signaling and adipocyte fatty acid metabolism. As this T_H_1 profile spreads through the bloodstream, other actively metabolic organs, such as the liver, muscle, and blood vessels, are affected by this deleterious condition and establish systemic inflammation, insulin resistance, and lipid dysregulation. Consequently, cardiovascular comorbidities develop, which, together with metabolic alterations, are already pathogenic features of SLE, exponentially increasing the severity, activity, and clinical deterioration of the disease. MiRs are involved in all substantial parts of the etiology, pathophysiology, and progression of both SLE and MetS, as they are critical regulators of gene expression of cytokines, adipokines, cell growth factors, and metabolic switches. Moreover, their expression and regulatory activity can be modified by conditions such as hypoxia and interleukins, impacting their tissue and circulating levels, making them potential specific and sensitive biomarkers for diagnosis, disease development prediction, and disease severity progression. Abbreviatures: SLE: systemic lupus erythematosus; MetS: metabolic syndrome; IR: insulin resistance; InsR: insulin receptor; IRS-1: insulin receptor substrate-1; IRS-2: insulin receptor substrate-1; Grb2: growth factor receptor-bound protein 2; SOS: son of sevenless protein; ApoB: apolipoprotein B; LDL-c: low-density lipoprotein cholesterol; HDL-c: high-density lipoprotein cholesterol; VLDL-c: very-low-density lipoprotein cholesterol; TNF-α: tumor necrosis factor-alpha; PAI-1: plasminogen activator inhibitor-1; IFN-I: interferon type I; CCL2: C-C motif chemokine ligand 2; IL-1: interleukin-1; IL-6: interleukin-6; IL-21: interleukin-21; IL-4: interleukin-4; IL-10: interleukin-10; IL-13: interleukin-13; IL-5: interleukin-5; IL-33: interleukin-33; iNKT: invariant natural killer T cells; Treg: regulatory T cells; Eos: eosinophils; ILC2; type 2 innate lymphoid cells; M2: macrophages type 2; M1: macrophages type 1; CLS: crown-like structures; CAVI: cardio-ankle vascular index; cIMT: carotid intima-media thickness; cfPWV: carotid–femoral pulse wave; miR: microRNA; mRNA: messenger RNA; Ago2: argonaute-2; miRISC: miRNA-induced silencing complex; UTR: untranslated region; T_H_1: T cell helper 1; T_H_2: T cell helper 2. Created with BioRender.com. Agreement number VW26L47W1D.

MetS is a group of different physiological abnormalities with a metabolic background; in SLE, there is a high prevalence of MetS with a higher probability of developing heart disease [93,94]. Among the efforts of multidisciplinary health teams, proposals for early markers for detection, markers to assess the severity of the disease, and even markers to observe the response to treatment stand out. However, the more markers are studied, the more knowledge needs to appear; mainly, it has been observed how inflammation has a leading role in mediating both pathologies and, to a large extent, the release of cytokines mediates this inflammation [95]. In Figure 1, we propose a set of assessments to better project the diagnosis and evolution of patients with SLE and MetS. Biomarkers include metabolic, immunometabolic, clinical, and miR parameters, which are dysregulated in these patients and are associated with disease severity.

In the clinical history of the disease, essential studies must be carried out on the patients, firstly, to define if the condition exists and, consequently, to determine the activity of the disease [96]. The constant stimulation of the immune system in SLE leads to the production of autoantibodies, the deposition of immune complexes, and the expression and secretion of cytokines. These cytokines are part of the activation of the cells of the immune system, which secrete more cytokines, creating a positive feedback cycle; this can cause the disease to become more severe at certain times [97]. Many of these cytokines also modulate their expression profile in MetS, and there is an exact association between these profiles and both diseases [98].

Few studies detail the presence or absence of MetS when evaluating cytokines or adipokines in SLE despite the relationship between both pathologies. Indeed, although the origin of inflammation is different, the mechanisms of execution of the immune system are similar.

Certainly, obesity has been considered a promoter of autoimmune diseases; in particular, the increase in fat mass has been associated with MetS and the inflammatory response, and it is located as an etiological factor of many diseases, including atherosclerosis, vascular events’ mortality, and TD2 [99,100]. However, the precise mechanisms by which it favors the progression of said pathologies still need to be established.

Obesity is the leading cause of developing CVD, which has produced a significant number of causes of death in patients with SLE [100]. That is why continuous surveillance of CVD risk is so important. In a certain way, it is not so common for patients with autoimmune diseases to carry out close surveillance with a cardiologist. However, now it has become a clinical necessity due to the various studies that associate the increased risk in these patients.

Finally, among the newest markers are miRs, which have been studied in various pathologies, such as detection, in response to treatment, and even as future therapies. The miRs have been evaluated from different perspectives, and, at a certain point, the large number of miRs that have been reported becomes overwhelming; however, between SLE and MetS, the associations made punctually between both pathologies are rare.

## 7. Conclusions

SLE is a complex pathology with different clinical moments throughout its course, during which various factors intervene in the disease’s severity and the appropriate immune response. On the other hand, MetS comprises metabolic alterations that, when present in a patient with SLE, potentiate the abnormal cellular imbalance and amplify the chances of exacerbation of the disease. SLE and MetS are closely connected so that factors in common such as diet, sex hormones, systemic and subclinical low-grade inflammation, treatment, T2D, IR, hyperlipidemia, cardiovascular abnormalities, and aberrant immune response are involved in the generation of markers that may be useful in daily clinical practice for the monitoring and selection of appropriate treatment. Metabolic profiles, proinflammatory molecules, adipokines, biophysical cardiovascular assessments, and miRs’ quantification can be considered as a set of assessments that, in association with clinical findings, could elucidate a biologic–clinical causality to improve diagnosis, follow-up, and treatment in patients with SLE and MetS.

## 8. Perspectives

We consider it essential to perform a set of evaluations that include immunometabolic, biophysical, and epigenetic biomarkers; however, it should be considered that most human studies have limitations such as sample size, genetic variability, sex, age, dietary habits, and treatment, among others. Although experimental studies have been essential to elucidate the pathophysiological processes in MetS and SLE by controlling the intervening variables, the extrapolation of the results has been limited on some occasions. Therefore, we consider the relevance of the identification and quantification of biomarkers that present clinical correlation, predictive value, and sensitivity and that are non-invasive. About miRs, which are specific biomarkers to be detected in early diagnoses of various diseases (i.e., carcinomas), an advantage to consider is the quantification of expression levels in biological samples of easy access (serum, ductal lavage, urine, fecal matter) compared to invasive procedures (biopsy, cerebrospinal fluid, tissue by surgical procedure). In addition, it is possible to associate its expression with diverse clinical findings, which is essential to highlight the biological–clinical causality that will help with the other clinical tools that will allow premature diagnoses and the establishment of more accurate prognoses.

## Figures and Tables

**Table 1:** SLE and MetS adipokine levels.

Adipokines ^1^	SLE	MetS	SLE and MetS	Ref.
Leptin	Increase	Increase	Increase with MetS	[45,57,58,59]
Adiponectin	Increase	Decrease	Decrease with MetS	[42,58,59]
Resistin	Increase	Increase	Increase with MetS	[42,58]
Chemerin	Increase (LN)	Increase	No data found	[50,60]
Visfatin	Increase (LN)	Increase	No data found	[51,54,61]
Omentin-1	Decrease	Decrease	No data found	[55,62]
Adipsin	Increase	Increase	No data found	[48,62]

^1^ Serum or plasma levels. Abbreviatures: LN: lupus nephritis.
